# Transcriptomic and Proteomic Approaches to Finding Novel Diagnostic and Immunogenic Candidates in *Pneumocystis*

**DOI:** 10.1128/mSphere.00488-19

**Published:** 2019-09-04

**Authors:** Taylor Eddens, Waleed Elsegeiny, David Ricks, Meagan Goodwin, William T. Horne, Mingquan Zheng, Jay K. Kolls

**Affiliations:** a UPMC Children’s Hospital of Pittsburgh, Pittsburgh, Pennsylvania, USA; b Center for Translational Research in Infection and Inflammation, Tulane School of Medicine, New Orleans, Louisiana, USA; Carnegie Mellon University

**Keywords:** *Pneumocystis*, diagnostics, life cycle, transcriptomics, vaccines

## Abstract

*Pneumocystis* pneumonia is the most common serious opportunistic infection in patients with HIV/AIDS. Furthermore, *Pneumocystis* pneumonia is a feared complication of the immunosuppressive drug regimens used to treat autoimmunity, malignancy, and posttransplantation rejection. With an increasing at-risk population, there is a strong need for novel approaches to discover diagnostic and vaccine targets. There are multiple challenges to finding these targets, however. First, *Pneumocystis* has a largely unannotated genome. To address this, we evaluated each protein encoded within the *Pneumocystis* genome by comparisons to proteins encoded within the genomes of other fungi using NCBI BLAST. Second, *Pneumocystis* relies on a multiphasic life cycle, as both the transmissible form (the ascus) and the replicative form (the trophozoite [troph]) reside within the alveolar space of the host. To that end, we purified asci and trophs from Pneumocystis murina and utilized transcriptomics to identify differentially regulated genes. Two such genes, *Arp9* and *Sp*, are differentially regulated in the ascus and the troph, respectively, and can be utilized to characterize the state of the *Pneumocystis* life cycle *in vivo*. *Gsc1*, encoding a β-1,3-glucan synthase with a large extracellular domain previously identified using surface proteomics, was more highly expressed on the ascus form of *Pneumocystis*. GSC-1 ectodomain immunization generated a strong antibody response that demonstrated the ability to recognize the surface of the *Pneumocystis* asci. GSC-1 ectodomain immunization was also capable of reducing ascus burden following primary challenge with Pneumocystis murina. Finally, mice immunized with the GSC-1 ectodomain had limited fungal burden following natural transmission of *Pneumocystis* using a cohousing model.

**IMPORTANCE** The current report enhances our understanding of *Pneumocystis* biology in a number of ways. First, the current study provided a preliminary annotation of the Pneumocystis murina genome, addressing a long-standing issue in the field. Second, this study validated two novel transcripts enriched in the two predominant life forms of *Pneumocystis*. These findings allow better characterization of the *Pneumocystis* life cycle *in vivo* and could be valuable diagnostic tools. Furthermore, this study outlined a novel pipeline of -omics techniques capable of revealing novel antigens (e.g., GSC-1) for the development of vaccines against *Pneumocystis*.

## Abstract (precis)

The current report enhances our understanding of *Pneumocystis* biology in a number of ways. First, the current study provided a preliminary annotation of the Pneumocystis murina genome, addressing a long-standing issue in the field. Second, this study validated two novel transcripts enriched in the two predominant life forms of *Pneumocystis*. These findings allow better characterization of the *Pneumocystis* life cycle *in vivo* and could be valuable diagnostic tools. Furthermore, this study outlined a novel pipeline of -omics techniques capable of revealing novel antigens (e.g., GSC-1) for the development of vaccines against *Pneumocystis*.

## INTRODUCTION

Pneumocystis jirovecii is an opportunistic fungal species responsible for infections that can cause fulminant pneumonia in immunocompromised individuals (1). HIV/AIDS patients in developing nations have a high incidence of *Pneumocystis* (PC) infection, in part because access to combination antiretroviral therapy (cART) and prophylactic trimethoprim-sulfamethoxazole (TMP-SMX) is limited (2, 3). However, even in regions of the developing world where access to cART and TMP-SMX is less limited, *Pneumocystis* remains the most common serious opportunistic infection in patients with HIV/AIDS (4–6). Furthermore, *Pneumocystis* has emerged in the non-HIV immunosuppressed population (7). Patients receiving novel therapeutics for hematologic malignancy, autoimmune disease, and posttransplantation rejection represent the groups whose risk of developing *Pneumocystis* is highest (8, 9). In addition to the increasing frequency of infection, non-HIV immunosuppressed patients with *Pneumocystis* pneumonia also have higher morbidity and mortality rates than HIV-positive patients (8, 10, 11).

The myriad of genetic, acquired, and induced immunosuppressive states capable of conferring susceptibility to *Pneumocystis*, while undesirable clinically, are instructive in understanding the protective immunologic mediators against *Pneumocystis* (12). The HIV/AIDS epidemic clearly illustrated that CD4^+^ T cells were the central orchestrators in the immune response against *Pneumocystis* (13). CD4^+^ T cells are responsible for coordinating the innate immune response during *Pneumocystis* infection, as protective eosinophils are recruited to the lung of mice early in the course of disease (14). However, CD4^+^ T cells also facilitate adaptive immunity with regard to production of anti-*Pneumocystis* IgG, which can provide protection even in the setting of passive transfer to an immunodeficient mouse (15). Antibodies can eliminate *Pneumocystis* either through opsonic phagocytosis in collaboration with macrophages or through complement-mediated nonopsonic killing (16, 17). Generation of protective anti-*Pneumocystis* antibodies has also been utilized in preclinical models of potential vaccine development (18).

One of the many hurdles encountered in attempts to construct a protective vaccine against *Pneumocystis* is the multiphasic life cycle utilized by the fungus (19). The trophozoite (troph) form of *Pneumocystis* ranges in size from 2 to 8 μm and replicates both asexually and sexually (20, 21). Asexual reproduction occurs through binary fission, while sexual reproduction occurs when two trophs conjugate via pheromone receptor signaling and fuse (19, 20). The fused trophs form a single diploid early sporocyte, which divides using meiosis and then mitosis to generate eight ascospores housed within the ascus or cyst form (19, 22). The ascus contains a thick β-1,3-glucan-rich shell and ultimately perpetuates the life cycle by releasing the eight ascospores as trophs (19, 23, 24). The ascus appears to be the transmissible form of *Pneumocystis*, as treatment with echinocandin antifungals inhibits β-1,3-glucan synthase, depletes the asci, and prevents aerosolized passage of the fungus between animals (25, 26). The ascus form also preferentially stains with Grocott’s methenamine silver (GMS) stain and thus is routinely observed during the diagnostic workup of *Pneumocystis* (27). The fluidity of the *Pneumocystis* life cycle can present a challenge for diagnosis, however, as at least one case of GMS-negative *Pneumocystis* has been described previously (28).

We hypothesized that vaccine development could be enhanced by targeting antibody-generating antigens enriched on the surface of various *Pneumocystis* life forms. RNA sequencing of separated asci and trophs revealed several novel life form-enriched transcripts. One such transcript, *Gsc1*, which we had shown previously to have a predicted ectodomain, was found in higher abundance in the ascus form by RNA sequencing and was depleted following micafungin treatment *in vivo*. Furthermore, immunization with the conserved ectodomain of GSC-1 was capable of reducing ascus burden following primary challenge in CD4^+^ T-cell-depleted mice. Finally, GSC-1 ectodomain immunization limited the burden following transmission of *Pneumocystis* to CD4^+^ T-cell-depleted mice in a cohousing model of infection.

## RESULTS

### Transcriptomic analysis of separated asci and trophs identifies differentially regulated diagnostic and vaccine targets.

**FIG 1:**
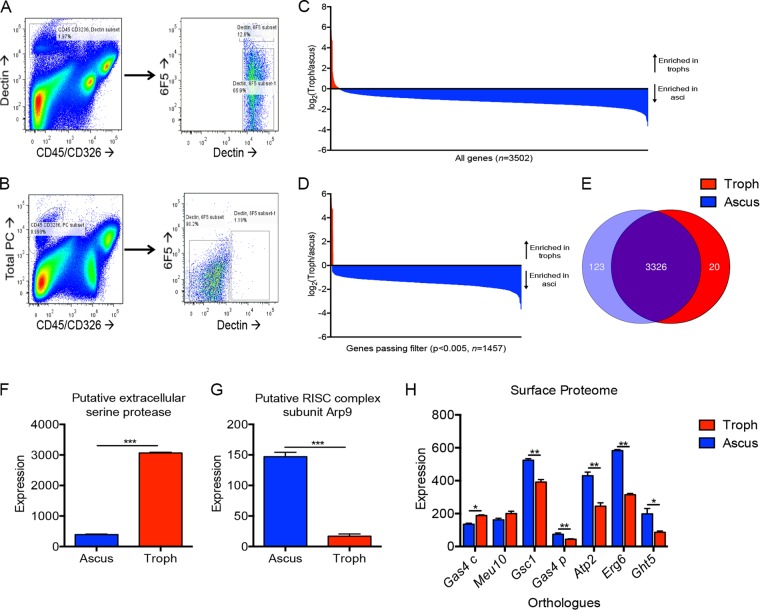
Separation of asci and trophs identifies differentially regulated transcripts. (A) Flow cytometric analysis on sorted asci and trophs. Asci were gated as CD45^−^ CD326^−^ Dectin-1^+^ and sorted accordingly (left). Asci were also variable with respect to 6F5 (right). (B) Trophs, isolated from BAL fluid of *Rag2^−^*^/^*^−^Il2rg^−^*^/^*^−^* mice treated with micafungin, were gated as CD45^−^ CD326^−^ PC^+^ (left). Trophs were Dectin-1 negative (right). Total *Pneumocystis* was gated using conjugated antisera (left). Asci were then labeled as Dectin-1:Fc positive (right), while trophs were Dectin-1 negative (bottom). (C) RNA sequencing analysis showing all expressed genes. Expression is shown as the log_2_ of troph expression (red) divided by ascus expression (blue). (D) Genes with significantly differential expression as measured by a *t* test performed with Benjamini and Hochberg correction (*P* < 0.005). (E) Genes expressed only in the ascus (*n* = 123) or troph (*n* = 20) as filtered by a quality value of 10. (F) A putative extracellular serine protease showed increased expression in the troph (*P* < 0.001). (G) A putative RISC subunit, Arp9, showed increased expression in the ascus (*P* < 0.001). (H) Differential levels of RNA expression of the seven genes previously identified via surface proteomics (*, *P* < 0.05; **, *P* < 0.01).

We had previously utilized surface biotinylation as an unbiased approach to identify extracellular proteins on whole *Pneumocystis* organisms and found seven novel antigenic targets (29). We next sought to classify the specific *Pneumocystis* life form expressing each protein. To that end, we isolated *Pneumocystis* from the bronchoalveolar lavage (BAL) fluid of a *Rag2^−^*^/^*^−^Il2rg^−^*^/^*^−^* mouse and sorted the asci from the trophs using flow cytometry (Fig. 1A). Asci were sorted by gating on CD45− CD326^−^ and Dectin-1:Fc fusion protein positivity, which we have demonstrated previously binds with high affinity to β-1,3-glucan (23) (Fig. 1A). Asci were also variable with respect to 6F5 (an anti-Kex1 monoclonal antibody), although all Dectin-positive organisms were sorted (Fig. 1A). Trophs, however, were isolated from the BAL fluid from a *Rag2^−^*^/^*^−^Il2rg^−^*^/^*^−^* mouse treated with micafungin, an echinocandin antifungal which selective depletes asci (25) (Fig. 1B). Trophs were identified by CD45^−^ CD326^−^, positivity of PC antisera, and were Dectin-1 negative by flow cytometry (Fig. 1B). Separation via flow cytometry resulted in enrichment for *Pneumocystis* transcripts compared to mouse transcripts (see Fig. S1 in the supplemental material). RNA was isolated from both separated asci and trophs and sequenced. Transcriptomic analysis revealed 95 genes upregulated in trophs, while 3,407 genes were more highly expressed in the asci with raw analysis (Fig. 1C). However, 16 genes were significantly upregulated in trophs, while 1,441 genes had significantly increased expression in the asci (Fig. 1D). Few genes had true differential expression, as only 123 genes had detectable transcription in the ascus, but not the troph (Fig. 1E). Likewise, only 20 genes were troph-specific with no transcription in the ascus (Fig. 1E). Interestingly, nine troph-specific transcripts corresponded to mitochondrial genes.

10.1128/mSphere.00488-19.1 FIG S1 Verification and enrichment of *Pneumocystis* transcripts by real-time PCR following sorting of various populations. Relative expression levels of mitochondrial large-subunit (mtLSU) ribosomal RNA (normalized to *Hprt* expression levels) are shown for the sorted *Pneumocystis* population, demonstrating an enrichment of fungus postsorting compared to the presorting value. Download FIG S1, PDF file, 0.04 MB. Copyright © 2019 Eddens et al. 2019 Eddens et al. This content is distributed under the terms of the Creative Commons Attribution 4.0 International license.

Using these transcriptomic data, we next sought to identify life form enriched target genes. A putative extracellular serine protease (*Sp*, PNEG_02319) had a high relative abundance comparative to other transcripts and was 8-fold more highly expressed in trophs (Fig. 1F). Contrastingly, a putative RNA-induced silencing complex (RISC) subunit gene, *Arp9* (PNEG_01343), showed significantly increased expression in the ascus life form compared to the troph (Fig. 1G). Following identification of troph- and ascus-enriched transcripts, we next queried the transcriptomic database for expression of the seven proteins previously identified by unbiased surface proteomics (29). Five proteins, *Gsc1*, *Gas4* (Schizosaccharomyces pombe homolog), *Atp2*, *Erg6*, and *Ght5* were significantly upregulated in the ascus (Fig. 1H). *Meu10* and *Gas4* (Saccharomyces cerevisiae homolog), however, had modest increases in the troph life form (Fig. 1H).

**FIG 2:**
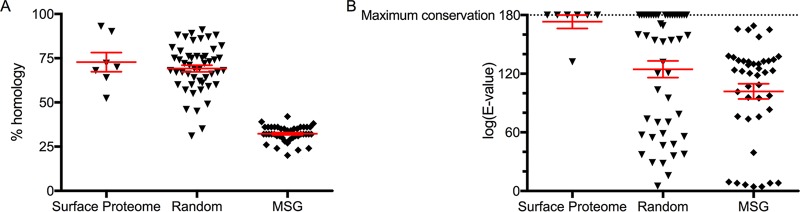
Conservation of surface proteomic targets compared to major surface glycoproteins and random proteins. (A) Percent homology between protein sequences of Pneumocystis murina surface proteomic targets, random proteins, and major surface glycoproteins (MSG) compared to Pneumocystis jirovecii sequences as determined by NCBI BLAST algorithm. (B) The absolute value of the log(E value) from comparisons between the described proteins above, again calculated using the NCBI BLAST algorithm, demonstrating a higher degree of conservation for surface proteomics targets than for random or MSG proteins.

The surface protein targets identified by proteomics also appear to be more highly conserved than major surface glycoproteins (MSG), a surface protein in *Pneumocystis* known to undergo antigenic variation and variable expression (30–32). While the *Pneumocystis* genome and thus the putative proteome have been sequenced, it largely remains unannotated, limiting the ability to make specific queries or comparisons (33). After a preliminary annotation of the complete *Pneumocystis* genome (see Table S1 in the supplemental material), surface proteins were found to be more conserved in both homology and E value using NCBI BLAST algorithm compared to MSG proteins (Fig. 2). Additionally, compared to 50 random non-MSG proteins, the surface proteome was highly conserved.

10.1128/mSphere.00488-19.4 TABLE S1 Preliminary annotation of the complete Pneumocystis murina genome. Download Table S1, XLSX file, 0.3 MB. Copyright © 2019 Eddens et al. 2019 Eddens et al. This content is distributed under the terms of the Creative Commons Attribution 4.0 International license.

### Ascus depletion *in vivo* reduces ascus-enriched transcript levels.

**FIG 3:**
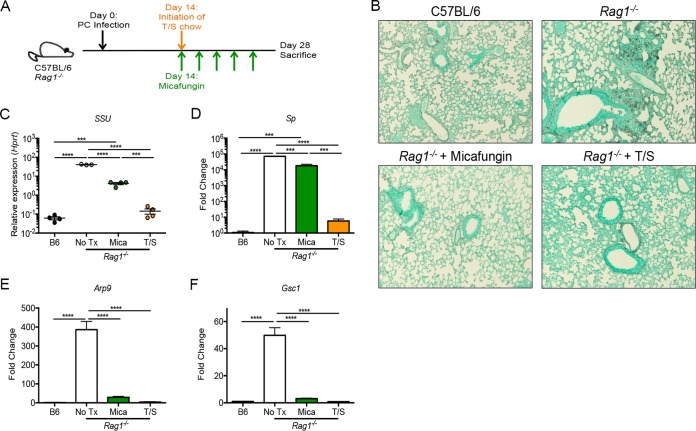
Depletion of asci *in vivo* reduces expression of *Arp9* and *Gsc1*. (A) *Rag1^−^*^/^*^−^* mice were infected with *Pneumocystis* and treated with either trimethoprim-sulfamethoxazole chow (T/S) or micafungin at day 14. C57BL/6 mice were infected and left untreated. (B) Representative images (100×) of GMS staining of the left lung at 28 days postinfection. (C to F) Small-subunit RNA (*SSU*) (C), *Sp* (D), *Arp9* (E), and *Gsc1* (F) gene expression at day 28 postinfection, showing selective depletion of ascus-enriched transcripts by micafungin treatment (*, *P* < 0.05; **, *P* < 0.01; ***, *P* < 0.001; ****, *P* < 0.0001 [by one-way ANOVA with Tukey’s multiple-comparison test]).

To further evaluate *Arp9* and *Sp* as life-form-specific markers *in vivo*, *Rag1^−^*^/^*^−^* mice were treated with either the ascus-depleting antifungal micafungin or trimethoprim-sulfamethoxazole (TMP-SMX), which eliminates both life forms (Fig. 3A). The ascus burden, as measured by Grocott’s methenamine silver (GMS) staining positivity, was depleted in *Rag1^−^*^/^*^−^* mice receiving either micafungin or TMP-SMX treatment to a level comparable to that seen with C57BL/6 mice (Fig. 3B). Untreated *Rag1^−^*^/^*^−^* mice, unsurprisingly, had detectable asci on histology (Fig. 3B). In contrast to the histologic findings, however, micafungin-treated *Rag1^−^*^/^*^−^* mice had small-subunit rRNA (*SSU*) transcript levels that were 100-fold higher than those seen with either the C57BL/6 or *Rag1^−^*^/^*^−^* mice receiving TMP-SMX treatment, suggestive of a persistent burden (Fig. 3C). Micafungin-treated *Rag1^−^*^/^*^−^* mice did show a 10-fold reduction in *SSU* compared to untreated *Rag1^−^*^/^*^−^* mice, consistent with partial depletion of the organism (Fig. 3C). Similarly to the *SSU* findings, micafungin-treated *Rag1^−^*^/^*^−^* mice showed a modest reduction in *Sp* expression compared to untreated *Rag1^−^*^/^*^−^* controls and *Sp* expression levels 1,000-fold higher than those seen with either the C57BL/6 mice or the TMP-SMX-treated *Rag1^−^*^/^*^−^* mice (Fig. 3D). Contrastingly, micafungin treatment reduced *Arp9* expression to the levels seen with the wild-type and TMP-SMX-treated mice (Fig. 3E). *Gsc1*, a surface protein enriched in asci, also showed significantly reduced expression following micafungin treatment (Fig. 3F).

### The GSC-1 ectodomain is highly conserved and is a natural antigen.

**FIG 4:**
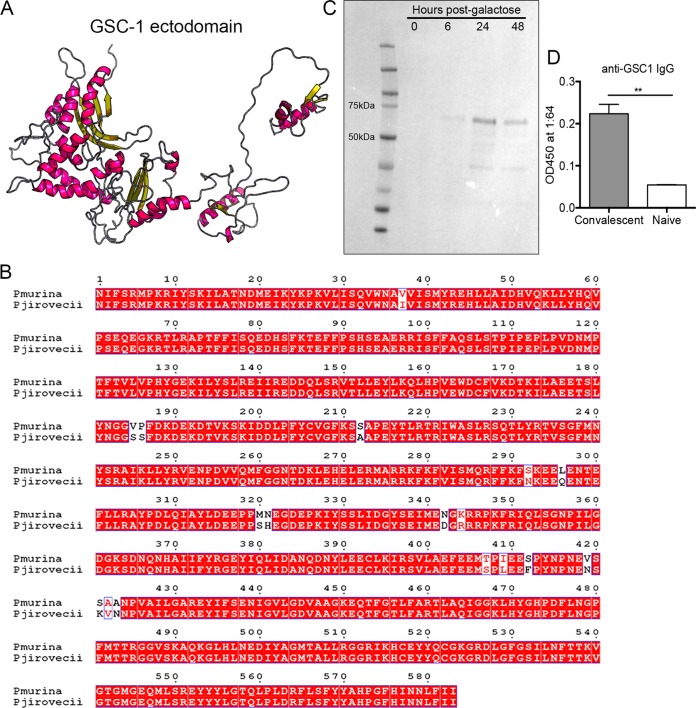
The GSC-1 ectodomain is highly conserved and immunogenic. (A) RaptorX prediction of the secondary protein structure of the GSC-1 ectodomain, showing α-helices and β-pleated sheets. (B) Alignment of the protein structures of the GSC-1 ectodomain in Pneumocystis murina and Pneumocystis jirovecii, showing few mismatches. (C) Recombinant GSC-1 ectodomain production in S. cerevisiae following induction with galactose-containing media. The predicted 71-kDa fragment was observed at highest concentration at 24 h postinduction. (D) Convalescent *Pneumocystis* sera recognize the GSC-1 ectodomain by ELISA, while naive sera do not (**, *P* < 0.01 [by Student's *t* test]).

As *Gsc1* expression is enriched in the infectious ascus form of *Pneumocystis*, we next sought to evaluate the antigenic potential of GSC-1. Pneumocystis murina GSC-1 has a 583-amino-acid extracellular domain (ectodomain) with predicted secondary structures, including α-helices and β-pleated sheets (Fig. 4A). However, any vaccine target would need to be conserved between the murine form of *Pneumocystis* and Pneumocystis jirovecii, the infectious species in humans. Pneumocystis jirovecii GSC-1 likewise has a predicted 583-amino-acid ectodomain (Fig. S2). The Pneumocystis murina and Pneumocystis jirovecii GSC-1 ectodomains have 98% identity at the amino acid level, demonstrating a high level of conservation across species (Fig. 4B). The *Gsc1* ectodomain was then cloned from Pneumocystis murina cDNA, and recombinant GSC-1 ectodomain was expressed using a galactose-inducible Saccharomyces cerevisiae system (Fig. 4C). A band was detectable at the predicted 71-kDa weight and was most prominently observed 24 h after galactose induction (Fig. 4C). The recombinant GSC-1 was then purified using a His column (Fig. S3). Interestingly, convalescent-phase serum samples from mice previously infected with *Pneumocystis* recognized the recombinant GSC-1 ectodomain by enzyme-linked immunosorbent assay (ELISA) (Fig. 4D). These data suggest that GSC-1 is a natural immunogen following primary challenge with *Pneumocystis*.

10.1128/mSphere.00488-19.2 FIG S2 Predicted topology of the Pneumocystis jirovecii GSC-1 demonstrating a large (583-amino-acid) ectodomain. Download FIG S2, TIF file, 0.2 MB. Copyright © 2019 Eddens et al. 2019 Eddens et al. This content is distributed under the terms of the Creative Commons Attribution 4.0 International license.

10.1128/mSphere.00488-19.3 FIG S3 Purification of recombinant GSC-1 ectodomain. Western blotting demonstrated the presence of V5-tagged GSC-1 ectodomain in the yeast culture (C), yeast lysate (L), and both the supernatant (S) and the pellet (P), as well as in the elution buffer (E). No GSC-1 was observed in the flowthrough (FT) or during the various wash steps (W). Download FIG S3, TIF file, 0.4 MB. Copyright © 2019 Eddens et al. 2019 Eddens et al. This content is distributed under the terms of the Creative Commons Attribution 4.0 International license.

### GSC-1 ectodomain immunization reduces the ascus burden, but not the total pneumocystis burden, following primary challenge.

**FIG 5:**
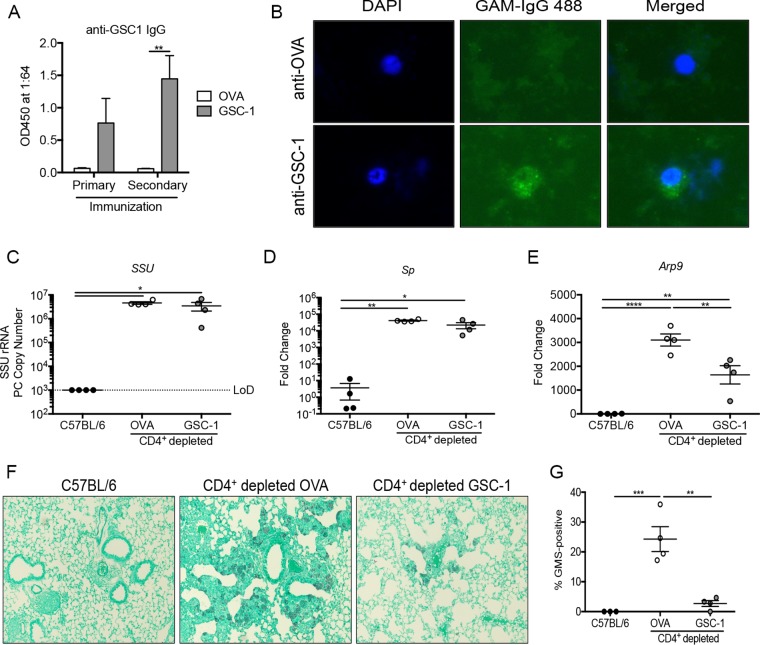
GSC-1 ectodomain immunization reduces ascus burden following *Pneumocystis* challenge. (A) Primary and secondary immunizations with the GSC-1 ectodomain, but not OVA, conjugated with alum generated anti-GSC-1 IgG (**, *P* < 0.01 [by Student's *t* test]). (B) Sera from GSC-1-immunized mice recognized the ascus form of *Pneumocystis* by immunofluorescent staining. (C) Following immunization, mice were subjected to CD4^+^ depletion and challenged with *Pneumocystis*. The *SSU* gene copy number was significantly elevated in both OVA-immunized and GSC-1-immunized mice. (D) *Sp* expression was significantly increased in both OVA-immunized and GSC-1-immunized mice. (E) *Arp9* expression was significantly reduced following GSC-1 immunization. (*, *P* < 0.05; **, *P* < 0.01; ****, *P* < 0.0001 [by one-way ANOVA with Tukey’s multiple-comparison test]). (F) GMS staining of the left lung at day 28 postinfection showing a reduced ascus burden in GSC-1-immunized mice. (G) Quantification of GMS positivity using ImageJ software showing significantly reduced fungal burden in GSC-1-immunized mice (**, *P* < 0.01; ***, *P* < 0.001 [by one-way ANOVA with Tukey’s multiple-comparison test]).

To assess the immunogenicity of the GSC-1 ectodomain *in vivo*, C57BL/6 mice were immunized either with the GSC-1 ectodomain or with ovalbumin (OVA) complexed with alum. At 2 weeks after primary vaccination, mice received a second immunization with the respective proteins. Mice receiving GSC-1 ectodomain immunization had a significant increase in anti-GSC1 IgG compared to OVA controls (Fig. 5A). In addition to recognizing the recombinant GSC-1 ectodomain, anti-GSC1 sera stained the surface of Pneumocystis murina asci, while anti-OVA sera did not (Fig. 5B).

To examine protective immunity, following two immunizations, the mice were rendered immunodeficient through administration of an anti-CD4 monoclonal antibody (GK1.5) to deplete CD4^+^ T cells and were subsequently challenged with *Pneumocystis*. At 4 weeks postchallenge, both the OVA-immunized mice and the GSC-1 ectodomain-immunized mice showed increased fungal burdens as assayed by *Pneumocystis SSU* quantitative reverse transcriptase PCR (RT-PCR) compared to C57BL/6 mice with intact immune systems (Fig. 5C). Likewise, both OVA-immunized mice and GSC-1 ectodomain-immunized mice showed increased *Sp* expression compared to C57BL/6 controls (Fig. 5D). However, the GSC-1 ectodomain-immunized mice showed a significant decrease in *Arp9* expression compared to the OVA-immunized mice (Fig. 5E). Consistent with the decreased ascus burden, fewer GMS-positive organisms were observed in GSC-1 ectodomain-immunized mice than in OVA-immunized mice (Fig. 5F). Quantification of the GMS-positive staining also demonstrated significant reduction in GSC-1-immunized mice (Fig. 5G). Additionally, inflammation in the lung following GSC-1 immunization was decreased on histology (Fig. 5F).

### GSC-1 ectodomain immunization limits burden in model of pneumocystis transmission.

**FIG 6:**
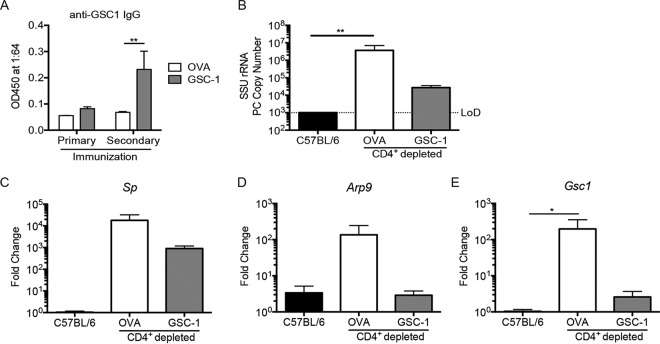
GSC-1 ectodomain immunization reduces *Pneumocystis* burden following natural transmission. (A) Primary and secondary immunizations with the GSC-1 ectodomain conjugated to alum generated anti-GSC-1 IgG (**, *P* < 0.01 [Student's *t* test]). (B) Following immunization, mice were subjected to CD4^+^ depletion and cohoused with an infected *Rag2^−^*^/^*^−^Il2rg^−^*^/^*^−^* mouse for 4 weeks. OVA-immunized mice, but not GSC-1-immunized mice, had increased *SSU* copy numbers compared to C57BL/6 controls (**, *P* < 0.01 [by one-way ANOVA with Tukey’s multiple-comparison test]). (C to E) Gene expression of *Sp* (C), *Arp9* (D), and *Gsc1* (E) demonstrating diminished ascus signal in GSC-1-immunized mice. (*, *P* < 0.05 [by one-way ANOVA with Tukey’s multiple-comparison test]).

As GSC-1 ectodomain immunization appeared to reduce the ascus form of *Pneumocystis*, we next evaluated the ability of GSC-1 ectodomain immunization to prevent natural transmission of *Pneumocystis* using a cohousing model (34, 35). In that evaluation, mice receiving GSC-1 ectodomain immunization generated anti-GSC1 IgG responses (Fig. 6A). The immunized mice were then subjected to CD4^+^ T-cell depletion and cohoused with a *Pneumocystis*-infected *Rag2^−^*^/^*^−^Il2rg^−^*^/^*^−^* mouse carrying a high fungal burden. Mice receiving OVA immunization had a significantly higher burden than C57BL/6 mice (Fig. 6B). GSC-1 ectodomain-immunized mice, however, had restricted fungal growth in the lung compared to OVA-immunized mice (Fig. 6B). While immunization slightly reduced *Sp* expression, both the OVA-immunized mice and GSC-1 ectodomain-immunized mice showed a more than 1,000-fold increase in *Sp* expression compared to wild-type controls (Fig. 6C). However, GSC-1 ectodomain-immunized mice showed reductions in ascus-enriched *Arp9* and *Gsc1* transcripts compared to OVA-immunized mice (Fig. 6D and E).

## DISCUSSION

*Pneumocystis* utilizes a multiphasic life cycle involving the propagation of asci and trophs within the alveolar space of the host (19, 27). Therefore, understanding the molecular signatures expressed by both life forms would greatly enhance our understanding of the microbiology of *Pneumocystis*, as well as highlight novel diagnostic and therapeutic strategies. Our data demonstrate that the transcriptome of sorted asci contains 123 unique transcripts whereas the enriched troph population contains 20 unique transcripts. The ascus form, however, had increased transcript abundance for the vast majority of transcripts. While this may represent a true enrichment in transcription due to differing metabolic states and profiles, it is also possible that the structure of the ascus itself mediates increased transcript abundance. The ascus houses up to eight ascospores; thus, the majority of asci contain 8 “contents” (8C) of DNA (22). Trophs have a broader range of DNA quantities (1C to 4C) (22). The transcriptional profile of the asci may therefore feature higher levels of transcription than that of the troph simply as a consequence of having more nuclei.

Importantly, despite the potential for an effect of DNA quantity, we were able to identify a troph-enriched transcript, *Sp*, and an ascus-enriched transcript, *Arp9*, by focusing on genes with striking differences in expression levels. Depletion of the ascus *in vivo* with micafungin treatment drastically reduced the GMS positivity of the lung histology and concomitantly reduced *Arp9* expression levels. *SSU*, a mitochondrial gene, and *Sp* were affected by micafungin treatment only modestly. These findings demonstrate a novel quantitative real-time PCR (qRT-PCR)-based approach to characterize the status of the *Pneumocystis* life cycle *in vivo*. Clinically, in addition to the already validated qRT-PCRs, a diagnostic test capable of providing a quantitative description of the troph-to-asci ratio could lead to the development of a personalized antifungal treatment (36, 37). For example, use of an echinocandin such as caspofungin may enhance organism clearance in a patient with a high ascus load but may be less effective in a case of troph-dominated pneumonia.

The *Pneumocystis* life cycle is likely very dynamic within the lung, depending on the particular host-pathogen interactions. With the advent of newer immunotherapies capable of treating autoimmunity, malignancy, and posttransplantation rejection, the range of immunosuppressed patients has greatly increased. For example, patients receiving corticosteroids, cyclophosphamide, and methotrexate as well as anti-tumor necrosis factor (TNF), anti-CD20, and anti-CD52 biological agents all have shown documented examples of increased susceptibility to *Pneumocystis* (8, 38–47). It is possible, therefore, that differing regimens of immunosuppression could provide niches favoring one life form over another. One such illustrative case occurred in a child receiving treatment for leukemia (28). During the course of chemotherapy, the patient developed GMS-positive *Pneumocystis* pneumonia, which, after subsequent diagnosis and initiation of therapy, became GMS negative despite escalating qRT-PCR burdens, suggestive of a trophic pneumonia (28).

Importantly, the patient with GMS-negative *Pneumocystis* pneumonia described above also had decreased expression of *Gsc1*, encoding a surface protein responsible for β-1,3-glucan synthesis (28, 48). GSC-1 has a large extracellular domain that is highly conserved with respect to the sequence of the homologous protein in Pneumocystis jirovecii, the infectious species in humans (29, 49). These two factors, coupled with the unbiased RNA sequencing approach demonstrating ascus-enrichment, made GSC-1 an attractive vaccine candidate. The GSC-1 ectodomain induces a robust antibody response upon natural infection and immunization. Furthermore, GSC-1 ectodomain immunization is capable of reducing ascus burden following *Pneumocystis* challenge and of limiting productive infection following natural transmission. The efficacy of GSC-1 as a therapeutic target may be limited, as the GMS-negative patient had worsening *Pneumocystis* pneumonia in the context of decreasing levels of *Gsc1* expression and ascus burden (28). However, targeting the asci for prevention of transmission appears to be a viable vaccination strategy (26). Therefore, the use of unbiased -omics techniques leading to the identification of the GSC-1 ectodomain as a vaccine target exemplifies a proof-of-principle pipeline for the discovery of novel antigens. The same pipeline—determining surface expression, determining the life form, and determining conservation—could therefore be applied to several antigens to create a multivalent vaccine targeting multiple life forms with great clinical potential.

## MATERIALS AND METHODS

### Mice.

Female 6-to-8-week-old C57BL/6 mice were ordered from the Jackson Laboratories and were maintained in the Rangos Research Building Animal Facility. All studies were approved and performed in accordance with the ethical guidelines of the University of Pittsburgh (Animal Welfare Assurance no. A3187.01) and the National Institutes of Health Office of Laboratory Animal Welfare. All animal experiments were performed in accordance with the recommendations in the Guide for the Care and Use of Laboratory Animals of the National Institutes of Health and the Institutional Care and Use Committee (protocols 14084327, 14084328, and 16027674). The Rangos Research Building Animal Facility is accredited by the Association for Assessment and Accreditation of Laboratory Animal Care (AAALAC).

### Reagents.

Mice were depleted of CD4^+^ T cells by weekly intraperitoneal injections of 0.3 mg of GK1.5 monoclonal antibody as previously described (14, 15, 50). For asci depletion, mice were treated with 3 mg of micafungin every 3 days via intraperitoneal injection. For treatment of *Pneumocystis*, mice were fed chow containing 1.2% sulfamethoxazole and 0.2% trimethoprim (catalog no. 1811319/5TYG; T.R. Last) for 2 weeks.

### Separation of asci and trophs.

Whole *Pneumocystis* organisms were isolated from the lungs of a *Rag2^−^*^/^*^−^Il2rg^−^*^/^*^−^* mouse infected with *Pneumocystis* for 8 weeks via bronchoalveolar lavage with 1-ml aliquots of phosphate-buffered saline (PBS). Following centrifugation and resuspension in PBS, *Pneumocystis* organisms were treated with 0.4 μg anti-CD16/CD32. Following a 15-min incubation, cells were treated with a peridinin chlorophyll protein (PerCP)-conjugated Dectin-1:Fc fusion protein made previously (23). Additionally, organisms were treated with anti-*Pneumocystis* antisera conjugated to e450, anti-kexin (6F5) conjugated to A700, anti-CD45 conjugated to fluorescein isothiocyanate (FITC), and anti-CD326 conjugated to FITC. Following sorting of asci, the organisms were added to TRIzol reagent (Life Technologies). Due to poor RNA quality of the isolated trophs, troph enrichment was performed using bronchoalveolar lavage of a *Rag2^−^*^/^*^−^Il2rg^−^*^/^*^−^* mouse infected with *Pneumocystis* for 6 weeks and treated with micafungin for 2 weeks as described above.

### RNA sequencing.

Total RNA was isolated from purified asci and trophs and used to generate mRNA sequencing libraries using an Illumina TruSeq stranded mRNA sample preparation kit. Following purification of poly(A)-containing mRNA molecules using poly(T) oligonucleotide-attached magnetic beads, the mRNA was fragmented into small pieces using divalent cations. The cleaved RNA fragments were then copied into first-strand cDNA using reverse transcriptase and random primers. Strand specificity was achieved by using dUTP in the second-strand marking mix, followed by second-strand cDNA synthesis performed using DNA polymerase I and RNase H. These cDNA fragments then were processed by the addition of a single “A” base and subsequent ligation of the adapter. The products were then purified and enriched with PCR to create the final cDNA library. The cDNA libraries were validated using Kapa Biosystems primer premix kit with Illumina-compatible DNA primers and a Qubit 2.0 fluorometer. Quality was examined using Agilent Tapestation 2200.The cDNA libraries were pooled at a final concentration of 1.8 pM. Cluster generation completed using cBot and 50-bp paired-read sequencing was performed on Illumina Genome Analyzer IIx.

### Analysis of gene expression.

Raw reads from Illumina Genome Analyzer IIx in fastq format were trimmed to remove adaptor/primer sequences. Trimmed reads were then aligned using BWA (version 0.5.9, settings aln -o 1 -e 10 -i 5 -k 2 -t 8) against the Pneumocystis murina genome (courtesy of the Broad Institute) in geneSifter Analysis Edition for Next Generation Sequencing (Geospiza, Seattle, WA). Additional alignment and postprocessing were done with Picard tools (version 1.58) and included local realignment and score recalibration to generate a finale genomic aligned set of reads. Reads mapping to the genome were characterized as exons, introns, or intergenic using the matched annotation for the genomic reference sequence. The remaining unmapped reads from the genomic alignment were then aligned to a splice reference created using all possible combinations of known exons followed by categorizing these as known or novel splice events. These aligned data were then used to calculate gene expression levels by computing the total of exon and known splice reads for each annotated gene to generate a count value per gene. For each gene, there was also a normalized expression value generated in the following two ways: (i) reads per mapped million (RPM), which is calculated by computing the count value and dividing it by the total number (in millions) of mapped reads; and (ii) reads per mapped kilobase per million (RPKM), which is calculated by dividing the RPM value by the kilobase length of the longest transcript for each gene. The RPM values are subsequently used for comparing gene expression across samples to remove the bias represented by the different numbers of reads mapped per sample. RPKM values were subsequently used for comparing the levels of expression of different genes to remove the bias represented by the different numbers of mapped reads and different transcript lengths. A quality score of 10 was used as a filter for removal of transcripts with low levels of or undetectable expression.

### *Pneumocystis* genome annotation.

A collection of all hypothetical Pneumocystis murina proteins was downloaded from the Broad Institute *Pneumocystis* sequencing project. Each hypothetical protein was analyzed using the NCBI BLAST algorithm, and the annotated orthologue shown to be closest to each protein was recorded. Each surface protein, each major surface glycoprotein, and 50 randomly selected proteins were then analyzed again with NCBI BLAST restricted to the taxa *Pneumocystis* (taxid: 4753), Saccharomyces cerevisiae (taxid: 4932), Schizosaccharomyces pombe (taxid: 4896), and percent identity and data weres were then recorded. The E value was graphed as the absolute value of the log(E value).

### Reverse transcriptase quantitative PCR.

Briefly, the right middle lobe of lung was homogenized in TRIzol reagent (Life Technologies). Following the addition of chloroform and centrifugation, RNA in the aqueous phase was precipitated in isopropanol. Following centrifugation, RNA was washed with 75% ethanol, pelleted once more, and resuspended in nuclease-free water. Following incubation at 55°C, RNA was quantified using a NanoDrop instrument and 500 ng of RNA was converted to cDNA using an iScript cDNA synthesis kit (Bio-Rad). SsoAdvanced qRT-PCR universal probe supermix and iQ SYBR green (Bio-Rad) were then used to quantify cDNA abundance. The primers used included the following: for the *Pneumocystis* small-subunit rRNA gene (*SSU*), the forward (F) primer was CATTCCGAGAACGAACGCAATCCT, the reverse (R) primer was TCGGACTTGGATCTTTGCTTCCCA, and the 6-carboxyfluorescein (FAM) probe was TCATGACCCTTATGGAGTGGGCTACA; for the serine protease gene (*Sp*), the F primer was AGTAGGTGTCTCGTCACATAAAG and the R primer was CTGGAAGGGTTGAGTATCATAGAG; for *Arp9*, the F primer was CACCTCAGCCAAGAACATTTG and the R primer was CGCGTTGCAAGTTCCTTATC; and for *Gsc1*, the F primer was ATTATGCGCCGGAATATGG, the FAM probe was GCAGATACATATGATCCTTACGGTGTTCC, and the R primer was ACTGAAGAGGACGCTGAT.

### Recombinant GSC-1 purification and immunization.

The ectodomain of GSC-1 was cloned from *Pneumocystis* cDNA using the following primers: forward, CACCATGAATATATTCTCAAGAATGCCGAAAAGGATTTATTC; reverse, AATAATAAAAAGATTATTGATATGAAATCCAGGATGAGC. Following amplification with Phusion polymerase (Thermo Fisher Scientific), the PCR product was purified using a QIAquick gel extraction kit. The GSC-1 was then inserted into a p-ENTR/d-TOPO vector kit (catalog no. K240020; Thermo Fisher Scientific) by incubation with a high-salt solution, and the vector was propagated in One Shot MAX Efficiency DH5-α Escherichia coli. Following isolation of plasmid, GSC-1 sequence insertion and orientation results were verified using M13-PCR primers and Sanger sequencing (GENEWIZ). Using a Gateway LR clonase kit (catalog no. 11791019; Thermo Fisher Scientific) to perform recombination, the GSC-1 ectodomain sequence was then inserted into yeast expression vector pYES-DEST52 (Invitrogen) and propagated in E. coli. Saccharomyces cerevisiae was grown to mid-log phase at 30°C in yeast extract-peptone-dextrose broth and transformed with GSC-1-pYES-DEST52 vector using an *S.c.* EasyComp transformation kit (Invitrogen). To assess protein production, yeast containing the GSC-1-pYES-DEST52 vector was grown overnight in SC minimal medium devoid of uracil and diluted to an optical density at 600 nm (OD_600_) of 0.4. The organisms were then added to SC minimal media containing 20% galactose induction and collected at 0, 6, 24, and 48 h postinduction. To isolate protein, the yeast was resuspended in a sodium phosphate buffer containing EDTA, glycerol, and protease inhibitors and subjected to vortex mixing in acid-washed glass beads (0.4 to 0.6 mm in diameter). Following lysis, supernatants were collected and added to 4× SDS-PAGE loading buffer and run on Mini-PROTEAN TGX Stain-Free precast gels (Bio-Rad). The proteins were then transferred onto a nitrocellulose membrane, blocked with Tris-buffered saline with Tween 20 (TBST) containing 5% milk, and probed overnight with anti-V5-HRP antibody (catalog no. R96125; Invitrogen). For large-scale protein purifications, 1-liter cultures of transformed yeast were grown in induction media, lysed using a BioSpec Beadbeater with 0.4-to-0.6-mm-diameter acid-washed beads (catalog no. 11079105; BioSpec), and purified using a Qiagen nickel-nitrilotriacetic acid (Ni-NTA) Superflow cartridge (catalog no. 30721) in a MasterFlex L/S digital pump system. Purification was assessed and imaged using a Chemi-Doc set with the stain-free gel setting. Following purification, a Millipore buffer exchange column was used to resuspend the purified protein in PBS and a bicinchoninic acid (BCA) assay was performed to determine the protein concentration. RaptorX protein prediction software was used to assess secondary structure (http://raptorx.uchicago.edu/StructurePrediction/predict/). Clustal Omega software and ESpirit software were used to generate alignment (51). Immunization was administered intraperitoneally with 150 μg of GSC-1 complexed in a 1:1 ratio with Imject alum (catalog no. 77161; Thermo Scientific) every 2 weeks.

### Gsc-1 ELISA.

Coating of 96-well plates was performed overnight at 4°C in a 9.2 pH carbonate buffer with 100 ng/well of recombinant GSC-1. Plates were washed 5 times with phosphate-buffered saline with Tween 20 (PBST) and blocked in PBST–5% milk for 2 h at room temperature. Serum was diluted 1:64 in PBST–5% milk and incubated overnight at 4°C. Plates were then washed 5 times with PBST and treated with 1:1,000 goat anti-mouse IgG horseradish peroxidase (HRP) (catalog no. 1030-05; Southern Biotech) diluted in blocking buffer. Following 1 h of incubation at room temperature, plates were washed 5 times with PBST, developed with TMB solution (BD OptEIA; catalog no. 555214), quenched with H_2_SO_4_, and read at 450 nm.

### *Pneumocystis* immunofluorescent staining.

Pneumocystis murina samples were fixed onto glass slides via heat fixation, followed by treatment with ice-cold methanol as previously described (23, 29). The slides were then washed with PBS and treated with PBS containing 5% milk for 14 min. Anti-GSC-1 serum was diluted 1:1,000 in PBS and added to the slide for 30 min. Following washes, the slides were stained with 1:1,000 goat anti-mouse IgG conjugated with DyLight 488 (Thermo Scientific). The slides were then washed and counterstained with 4′,6-diamidino-2-phenylindole (DAPI) (1:2,000) for 15 min. After three further washes, the slides were mounted with VectaMount AQ mounting media (Vector Laboratories) and visualized using ×630 magnification.

### *Pneumocystis* infection.

Pneumocystis murina was propagated in *Rag2^−^*^/^*^−^Il2rg^−^*^/^*^−^* double-knockout mice for 8 weeks. Following removal of the lungs, the lungs strained through a 70-μm-pore-size filter. Mice were then subjected to oropharyngeal inoculation with 2.0 × 10^5^ cysts as previously described (52, 53).

### Histology.

The left lobe of lung was insufflated with 10% formalin via injection in the left main stem bronchus. The lung was then sectioned and stained with hematoxylin and eosin (H&E) and Grocott’s methenamine silver (GMS) stain in the Children’s Hospital of Pittsburgh Histology Core. Image quantification was performed using ImageJ, with conversion of the GMS images to a red-green-blue (RGB) stack and quantification of black/dark pixels on the green filter.

### Cohousing model of *Pneumocystis* transmission.

Immunized female C57BL/6 mice were subjected to CD4^+^ T-cell depletion and then transferred to a cage containing a *Rag2^−^*^/^*^−^Il2rg^−^*^/^*^−^* mouse that had been infected with *Pneumocystis* 4 weeks prior. The infected *Rag2^−^*^/^*^−^Il2rg^−^*^/^*^−^* mouse was then rotated between experimental cages every week for 4 weeks until the mice were sacrificed.

### Statistics.

All data analyses were performed using GraphPad Prism version 6.0f. All data are presented as means ± standards error of the means (SEM). For transcriptomic analysis, a *t* test was performed with a Benjamini and Hochberg correction and an adjusted *P* value of less than 0.005 was used to determine significance. For studies containing two groups, a Student's *t* test was used to analyze significance. For studies containing three or more groups, ordinary one-way analysis of variance (ANOVA) was used with Tukey’s multiple-comparison test to analyze the data. A *P* value of <0.05 represented significance in all statistical analyses.

### Data availability.

The RNA sequencing data contained in this paper are publicly available through Gene Expression Omnibus accession number GSE136100.
