# Supplementary figures and images for: Transcriptomic and Proteomic Approaches to Finding Novel Diagnostic and Immunogenic Candidates in Pneumocystis
Source: mSphere. 2019 Sep 4;4(5):e00488-19. doi: 10.1128/mSphere.00488-19 (PMC6731532; doi:10.1128/mSphere.00488-19)

Supplemental Figure 1

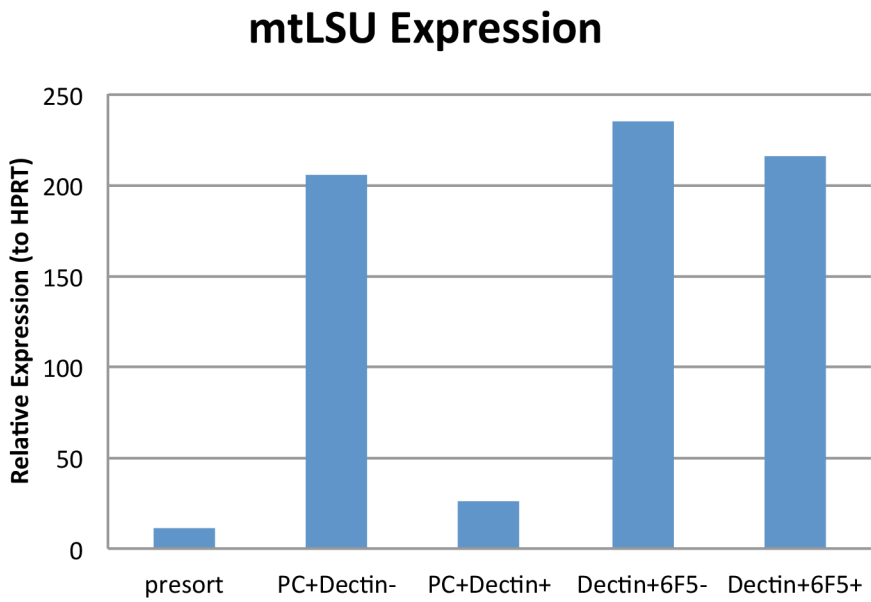

Supplement: FIG S1 [file mSphere.00488-19-sf001.pdf]

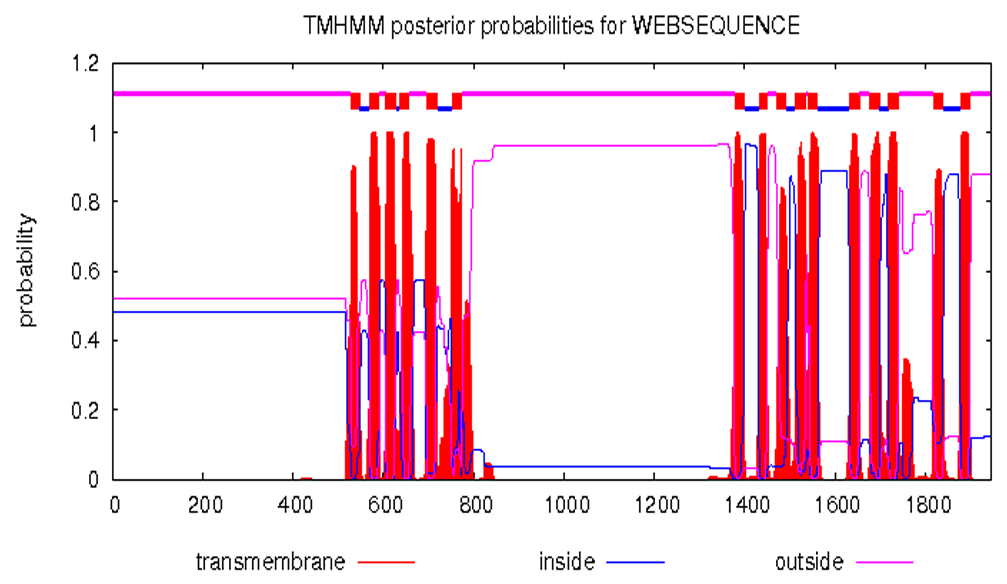

Supplement: FIG S2 [file mSphere.00488-19-sf002.tif]

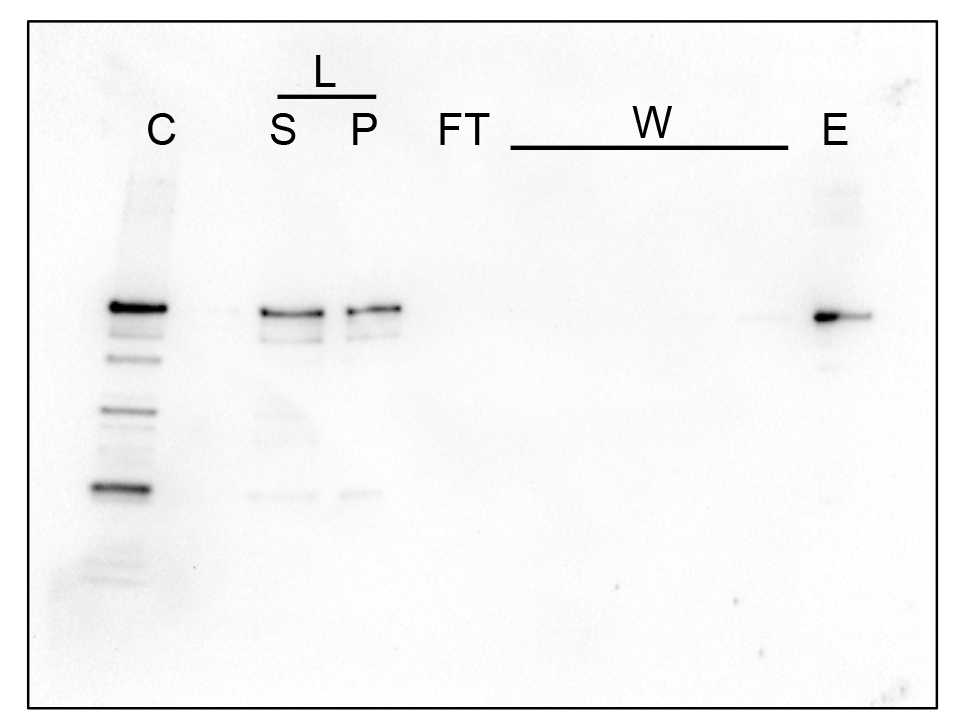

Supplement: FIG S3 [file mSphere.00488-19-sf003.tif]
